# AFF4 regulates osteogenic differentiation of human dental follicle cells

**DOI:** 10.1038/s41368-020-0083-9

**Published:** 2020-06-30

**Authors:** Qingyue Xiao, Yuning Zhang, Xingying Qi, Yaqian Chen, Rui Sheng, Ruoshi Xu, Quan Yuan, Chenchen Zhou

**Affiliations:** 1 State Key Laboratory of Oral Diseases & National Clinical Research Center for Oral Diseases & West China Hospital of Stomatology, Sichuan University, Chengdu, China; 2 State Key Laboratory of Oral Diseases & National Clinical Research Center for Oral Diseases & Department of Cariology and Endodontology, West China Hospital of Stomatology, Sichuan University, Chengdu, China; 3 State Key Laboratory of Oral Diseases & National Clinical Research Center for Oral Diseases & Department of Oral Implantology, West China Hospital of Stomatology, Sichuan University, Chengdu, China; 4 State Key Laboratory of Oral Diseases & National Clinical Research Center for Oral Diseases & Department of Pediatric Dentistry, West China Hospital of Stomatology, Sichuan University, Chengdu, China

**Keywords:** Stem-cell differentiation, Protein translocation

## Abstract

As a member of the AFF (AF4/FMR2) family, AFF4 is a transcription elongation factor that is a component of the super elongation complex. AFF4 serves as a scaffolding protein that connects transcription factors and promotes gene transcription through elongation and chromatin remodelling. Here, we investigated the effect of AFF4 on human dental follicle cells (DFCs) in osteogenic differentiation. In this study, we found that small interfering RNA-mediated depletion of *AFF4* resulted in decreased alkaline phosphatase (ALP) activity and impaired mineralization. In addition, the expression of osteogenic-related genes (*DLX5*, *SP7*, *RUNX2* and *BGLAP*) was significantly downregulated. In contrast, lentivirus-mediated overexpression of *AFF4* significantly enhanced the osteogenic potential of human DFCs. Mechanistically, we found that both the mRNA and protein levels of ALKBH1, a critical regulator of epigenetics, changed in accordance with AFF4 expression levels. Overexpression of *ALKBH1* in *AFF4*-depleted DFCs partially rescued the impairment of osteogenic differentiation. Our data indicated that AFF4 promoted the osteogenic differentiation of DFCs by upregulating the transcription of *ALKBH1*.

## Introduction

The integrity of dentition is of significant importance to oral health. Dentition defects can have adverse influences on oral functions, such as the vocalization, masticatory function and aesthetics, thus diminishing the patients’ quality of life.^1^ There is a high prevalence of periodontal problems, but tooth loss is not a good option. As a chronic infectious disease, periodontal disease is characterized by the inflammation of supporting tissues, such as gingiva, the periodontal membrane, the cementum and alveolar bone, resulting in loose or lost teeth.^2^ Dental prosthetic treatment plays a vital role in achieving mechanical and partial functional restoration. However, the ultimate goal of treatment is regenerating the damaged periodontal tissue to restore the original composition and function.^3^ With this goal in mind, periodontal tissue regeneration engineering provides a potential solution. The oral cavity contains rich sources of mesenchymal stem cells, and previous studies have shown that these stem cells have a superior ability to regenerate tissue in vitro, especially in terms of osteogenesis.^4^ Dental follicle cells (DFCs), which show excellent osteogenesis potential, can hopefully be a substitute for traditional marrow stem cells.^5^

The dental follicle, originating from the ectodermal mesenchyme, is a loose connective tissue that wraps around the developing tooth germ. During tooth development, periodontal supporting tissues originate from dental follicles and play a role in buffering, supporting, remodelling and regenerating the developing teeth.^6^ Furthermore, periodontal supporting tissues embody distinct embryonic features, such as high self-renewal capability, prominent proliferation potential, pluripotency, heterogeneity and multidirectional differentiation.^7^ The human dental follicle harbours a mass of stem cells that have a dramatic effect on periodontal tissue development and bone remodelling. DFCs can differentiate into osteoblasts, fibroblasts, neurons and adipocytes under the corresponding induction in vitro.^8^ DFCs possess several excellent characteristics: easy acquisition, minimal surgical trauma, excellent cell fusion ability and the ability to be stored a long time. Given their unique properties, DFCs have been regarded as promising seed cells for use in periodontal tissue regeneration engineering.^9^ Therefore, it is of great significance to explore the core target genes and regulatory mechanisms involved in periodontal tissue development, hopefully providing insights into strategies for periodontal tissue engineering.^10,11^

The differentiation of stem cells is a highly regulated process influenced by numerous factors at each step. As a critical step in gene expression, transcription is precisely regulated.^12^ As a central enzyme, RNA Polymerase II (RNA Pol II) is responsible for producing noncoding RNAs as well as pre-mRNAs.^13^ Mediated by RNA Pol II, gene transcription is a strictly controlled procedure that has four phases: transcription initiation, transcription pausing, transcriptional elongation and transcriptional termination. Among these processes, elongation is a significant rate-limiting stage that dictates the expression of the entire genome.^14^

The transcriptional elongation complex is a regulatory intermediate in the transcriptional elongation stage.^15^ After binding to the RNA polymerase, it transforms into a special spatial conformation that interacts with RNA, DNA and other elongation factors, completing signal reception and influencing transcription decisions during transcription pausing.^16^ With more research on the transcriptional elongation process, the super elongation complex (SEC) is receiving increasing attention for its communication with multitudinous target genes during transcriptional elongation, which is regarded as a significant rate-limiting stage.^17^ The SEC is promptly recruited to the target genes in response to differentiation signals, where it then releases the paused RNA Pol II to enable dynamic transcription.^18^ It has a positive regulating effect on transcriptional elongation, activating transcriptional activity and improving elongation efficiency. As a variable multiprotein complex, the SEC consists of several subunits: positive elongation factor b (P-TEFb), an AFF protein family member (scaffold protein), an ELL protein family member (the elongation factor of RNA Pol II) and a partner protein AF9 or ENL.^19^

Localized in the nucleus, the AFF protein family comprises four members: AFF1/2/3/4.^20^ In the SEC, significant emphasis is placed on AFF1 or AFF4, which serve as scaffold proteins that promote the assembly of other subunits and maintain the stability of the complex.^19,21^ AFF1 and AFF4 have been extensively implicated in the control of HIV transcription. At the RNA level, a previous analysis demonstrated that the effect of AFF4 is stronger than that of AFF1 on early HIV transcription.^22^ Furthermore, AFF1 and AFF4 are both critical epigenetic regulators, but they have different roles during the osteogenic differentiation of human mesenchymal stem cells (MSCs). Previous results show that AFF4 plays a pivotal role in stimulating MSC osteogenic differentiation.^23^

In this study, we tested the impact of AFF4 on the osteogenic differentiation of DFCs, and we analysed the underlying regulatory mechanism. Our results show that AFF4 can stimulate the osteogenic differentiation of DFCs by regulating *ALKBH1* expression.

## Results

### Expression of AFF4 in DFCs

**Fig. 1:**
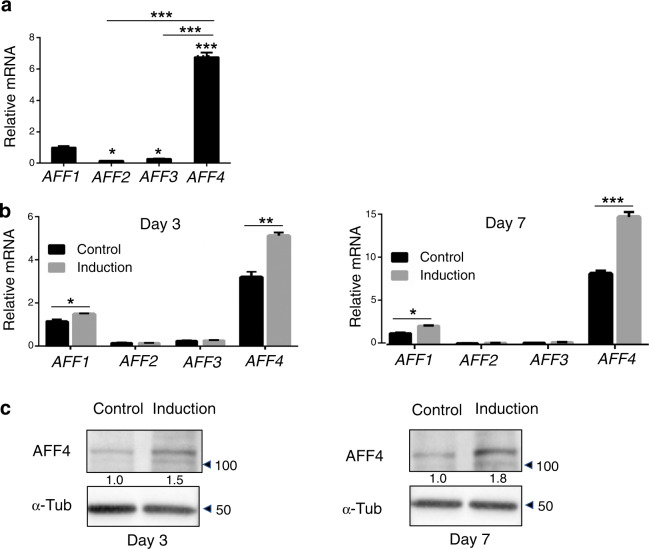
Expression of AFF4 in DFCs. **a** Quantitative RT-PCR shows that the expression of *AFF4* is the highest among AFF family members. *n* = 3. ***P < 0.001, and *P < 0.05. **b** Quantitative RT-PCR of AFF family members after osteogenic differentiation induction for 3 and 7 days. *n* = 3. ***P < 0.001, **P < 0.01, and *P < 0.05. **c** Western blot analyses of AFF4 after osteogenic differentiation induction for 3 and 7 days. The quantitation of the WB is shown

First, we investigated the expression of *AFF4* in DFCs. As shown by quantitative RT-PCR (qRT-PCR) analysis, among the AFF family members, the expression of *AFF4* was the highest. For other family members, *AFF1* had the next highest expression, while the expression of *AFF2* or *AFF3* was low (Fig. 1a).

We also conducted osteogenic induction on DFCs and analysed dynamic changes in expression of *AFF* family members. The results of qRT-PCR indicated that the expression of *AFF4* dramatically increased after induction, while there was no significant difference in *AFF2* or *AFF3*. (Fig. 1b). The analyses of western blot and semiquantification of AFF4 confirmed its higher expression after osteogenic induction relative to its levels before induction (Fig. 1c), suggesting that during the osteogenic differentiation of DFCs, AFF4 is a potential regulator.

### Depletion of *AFF4* decreases osteogenic differentiation of DFCs

**Fig. 2:**
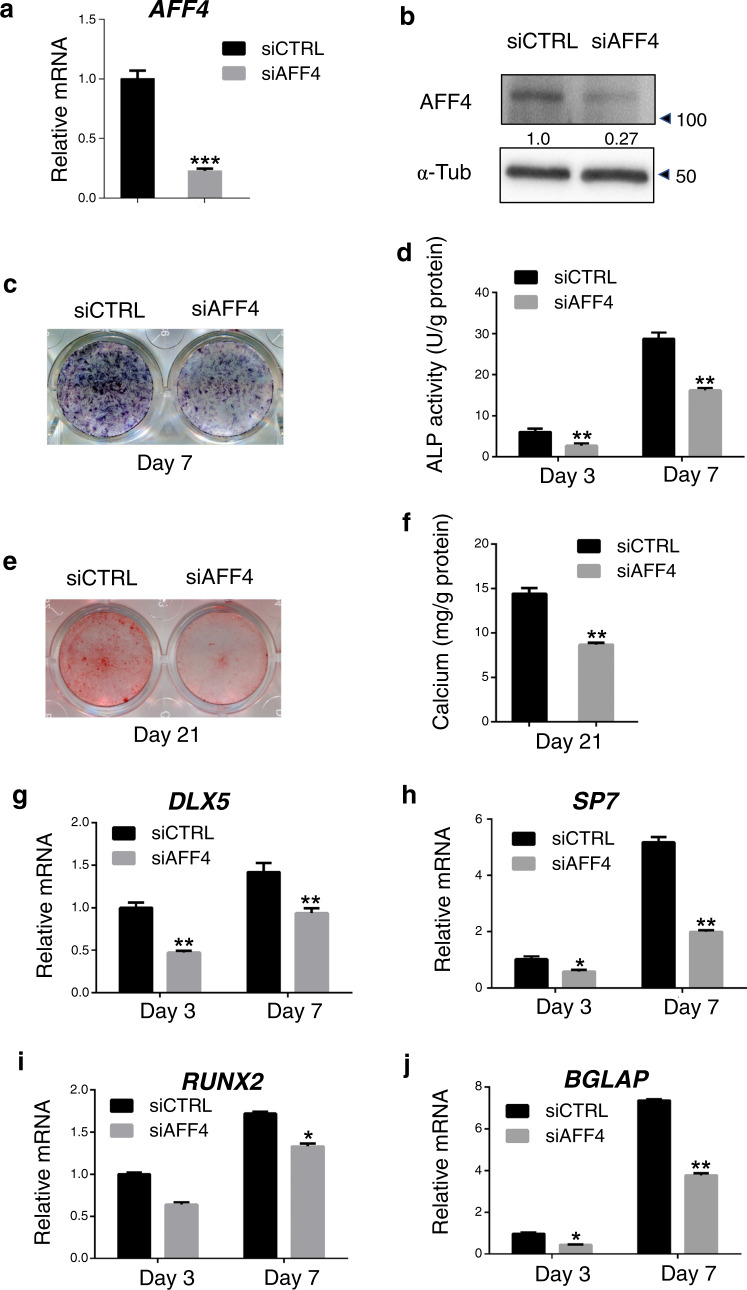
Depletion of *AFF4* decreases osteogenic differentiation of DFCs. **a** Quantitative RT-PCR showing the successful knockdown of *AFF4*. *n* = 3. ***P < 0.001. **b** Western blot analysis and semiquantification of AFF4. **c** Representative ALP staining images of DFCs. **d** Quantitative assessment of ALP activity. *n* = 5. **P < 0.01. **e** Representative images of ARS staining. **f** Quantitative assessment of calcium mineralization. *n* = 5. **P < 0.01. **g**–**j** Quantitative RT-PCR demonstrating the reduced expression of *DLX5*, *SP7* and *BGLAP*. *n* = 3. *P < 0.05, and **P < 0.01

To illustrate the importance of AFF4 in the osteogenic differentiation of DFCs, we knocked down *AFF4* in DFCs by treatment with a small interfering RNA (siRNA). Two days after transfection, qRT-PCR and western blot analyses were used to test the knockdown efficiency (Fig. 2a, b). We found that the staining of alkaline phosphatase (ALP), an essential marker of early osteogenic differentiation, was significantly reduced in the siRNA-mediated *AFF4* depletion group after osteogenic induction for 7 days (Fig. 2c). The results of quantitative ALP activity measurements also confirmed previous observations after osteogenic induction for 3 days and 7 days (Fig. 2d). Alizarin red S (ARS) staining and calcium deposition analysis were performed to investigate the impact of AFF4 on extracellular matrix mineralization. The results revealed that there was less ARS staining after *AFF4* depletion than there was in control cells (Fig. 2e). A quantitative measurement of calcium concentration, showed that it was also decreased as *AFF4* was depleted (Fig. 2f), indicating that AFF4 was significant in the mineralization process. In addition, we evaluated the expression of *DLX5*, *SP7*, *RUNX2* and *BGLAP*, and we found that these osteogenic-related genes were repressed in *AFF4*-depleted cells relative to their levels in control cells (Fig. 2g–j).

### Overexpression of *AFF4* promotes osteogenic differentiation of DFCs

**Fig. 3:**
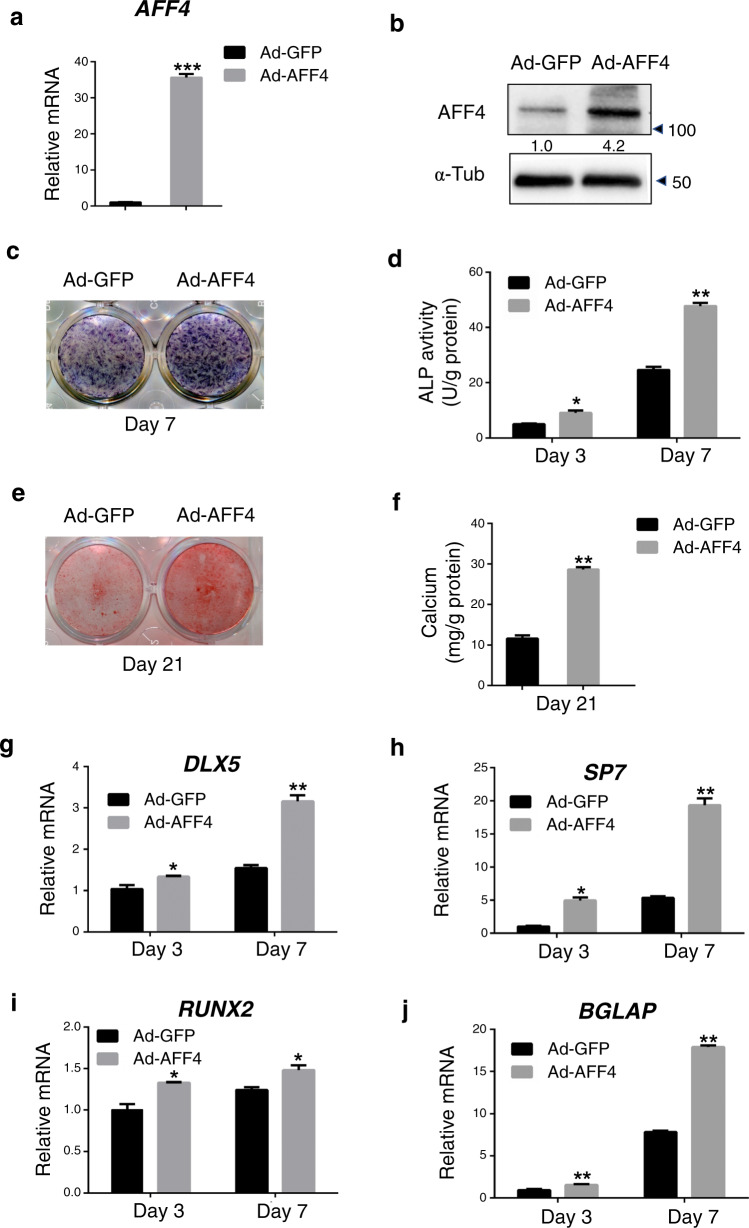
Overexpression of *AFF4* promotes osteogenic differentiation of DFCs. **a** Quantitative RT-PCR shows that the mRNA level of *AFF4* is significantly increased. *n* = 3. ***P < 0.001. **b** Western blot analysis and semiquantification of AFF4. **c** Representative ALP staining images of DFCs. Increased expression of AFF4 enhances the intensity of the staining. **d** Quantitative ALP activity assessment. *n* = 5. ***P < 0.001. **e** Representative ARS staining images of DFCs. **f** Quantitative calcium mineralization assessment. *n* = 5. **P < 0.01. **g**–**j** Quantitative RT-PCR displays the increased mRNA expression of *DLX*, *SP7* and *BGLAP*. *n* = 3. *P < 0.05 and **P < 0.01

We wondered whether the overexpression of *AFF4* could have an opposite effect to enhance osteogenic differentiation. DFCs were stably infected with lentivirus expressing GFP or *AFF4*. qRT-PCR and western blot analyses were performed to verify the overexpression efficiency (Fig. 3a, b). *AFF4* overexpression caused increased ALP staining and ALP activity after osteogenic induction (Fig. 3c, d). In addition, ARS staining and extracellular matrix mineralization were also markedly enhanced over that of control cells (Fig. 3e, f). Moreover, the overexpression of *AFF4* stimulated the expression of *DLX5*, *SP7* and *BGLAP*, as shown by qRT-PCR on osteogenic induction at day 3 and day 7 (Fig. 3g–j).

### AFF4 regulates the expression of ALKBH1

**Fig. 4:**
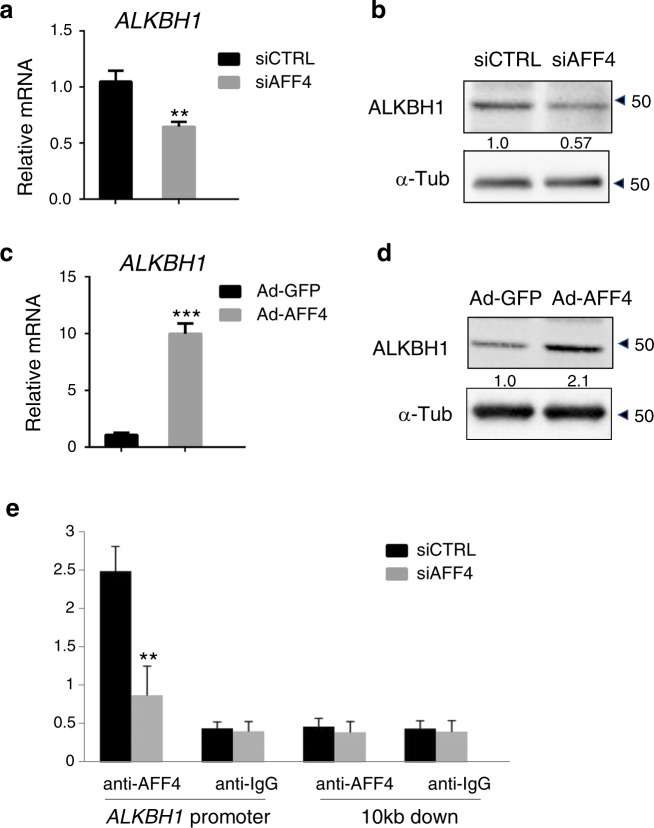
AFF4 regulates the expression of ALKBH1. **a**, **b** Quantitative RT-PCR, western blot and quantification of ALKBH1 after the depletion of *AFF4*. *n* = 3. **P < 0.01. **c**, **d** Quantitative RT-PCR, western blot and quantification of ALKBH1 after *AFF4* overexpression. *n* = 3. ***P < 0.001. **e** ChIP assay for AFF4 demonstrates that it binds to the promoter region of *ALKBH1*. *n* = 4. **P < 0.01

ALKBH1 is a critical epigenetic factor in the osteogenic differentiation of MSCs.^24^ Therefore, we wondered whether AFF4 could regulate the transcription of *ALKBH1*. First, we found that the expression of ALKBH1 was significantly reduced after *AFF4* depletion at both the mRNA and protein levels (Fig. 4a, b). Conversely, the overexpression of *AFF4* induced the expression of ALKBH1 (Fig. 4c, d). Next, an anti-AFF4 ChIP assay was conducted to further reveal the relationship between AFF4 and ALKBH1. We showed that AFF4 bound to the promoter region of *ALKBH1* and that the ChIP signal was largely abolished by *AFF4* depletion (Fig. 4e).

### Overexpression of *ALKBH1* partially rescues osteogenic differentiation of *AFF4*-deficient DFCs

**Fig. 5:**
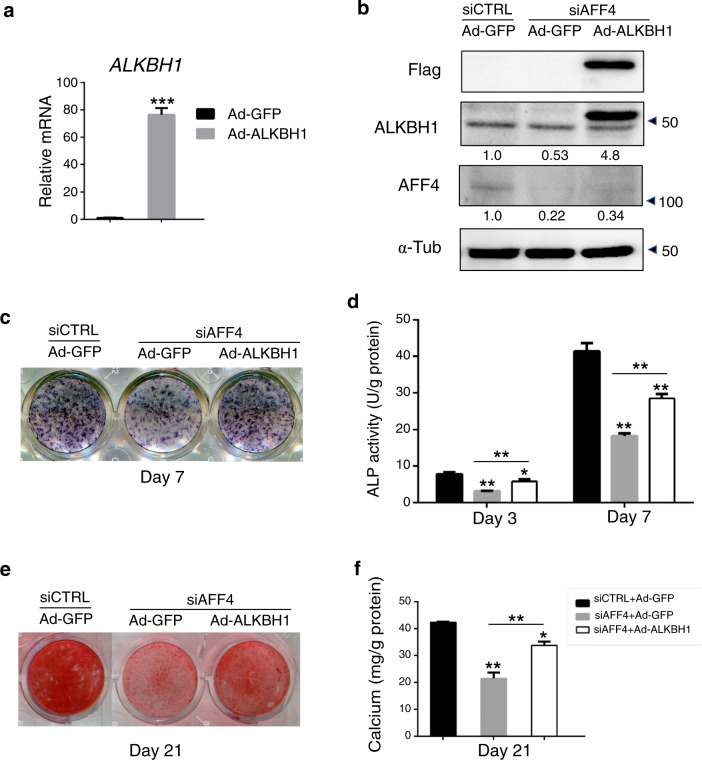
Overexpression of *ALKBH1* partially rescues osteogenic differentiation of *AFF4*-deficient DFCs. **a** The mRNA level of *ALKBH1*. *n* = 3. ***P < 0.001. **b** Western blot analyses of ALKBH1 and AFF4. Semi-quantifications of WB are shown. **c**, **d** Representative images of ALP staining and quantification of ALP activity. *n* = 5. *P < 0.05, and **P < 0.01. **e**, **f** Representative images of ARS staining and quantitative analyses of calcium mineralization. *n* = 5. *P < 0.05, and **P < 0.01

**Fig. 6:**
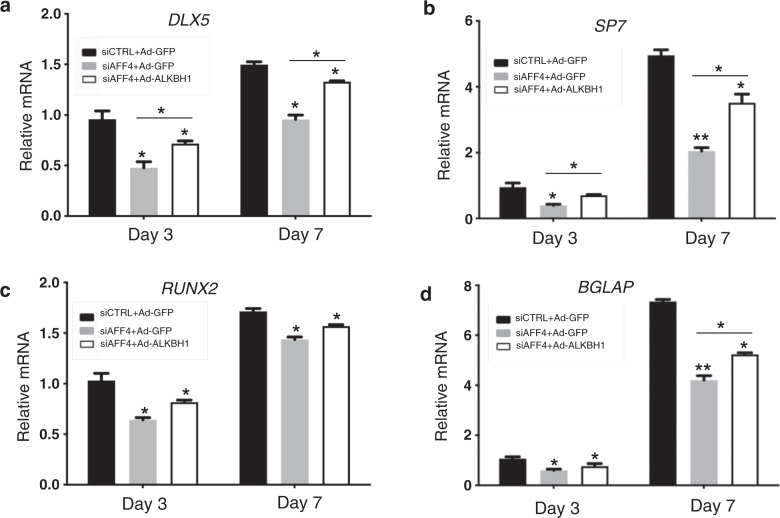
The expression of osteogenic-related genes in *ALKBH1*-overexpressing DFCs. **a**–**d** Quantitative RT-PCR reveals that overexpression of *ALKBH1* restores the blunted expression of *DLX*, *SP7*, *RUNX2* and *BGLAP*. *n* = 3. *P < 0.05, and **P < 0.01

To further elucidate the regulatory mechanism involving *ALKBH1*, rescue experiments were conducted by using a lentivirus to overexpress *ALKBH1* in *AFF4*-depleted DFCs. *ALKBH1* overexpression was confirmed to be efficient (Fig. 5a, b). Overexpression of *ALKBH1* in DFCs rescued the suppressed ALP staining and ARS staining caused by *AFF4* depletion (Fig. 5c, d). Quantitative measurement of ALP activity and of calcium concentrations also showed that overexpression of *ALKBH1* significantly abolished the inhibition of osteogenic differentiation caused by *AFF4* depletion (Fig. 5e, f). Finally, we performed qRT-PCR experiments, which revealed that *ALKBH1* overexpression restored the inhibited mRNA expression of *DLX5*, *SP7*, *RUNX2* and *BGLAP* (Fig. 6a–d).

## Discussion

Periodontal tissue regeneration is a key part of the treatment of periodontal disease.^3^ Pathological alveolar resorption is based on the negative balance of bone reconstruction with the decline of osteoblast function and the increase of osteoclast activity, which has an impact on the reconstruction of alveolar bone.^25^ The osteogenic differentiation of dental stem cells is a complicated activity, which follows the differentiation sequence of producing first osteogenesis progenitor cells, osteogenesis precursor cells and osteoblasts that eventually differentiate into bone cells. This process involves various types of intracellular and intercellular signal transmission and pathways, which constitute a complex signal transduction network.^26^

DFCs have the extraordinary ability to differentiate into different and diverse cell types. The periodontal tissues, such as the cementum, periodontal ligament and alveolar bone, all originate from dental follicles. The dental sac surrounding the tooth germ is the matrix where DFCs reside. DFCs, with the unique properties of stem cells, are important for the growth of tooth roots and tooth eruption. Various periodontal progenitor cells originate from the dental follicle and are associated with the construction of periodontal tissue.^8,12^

Dental follicle cells can differentiate into multiple cell types, forming periodontal ligament, cementum and alveolar bone during the development of periodontal tissue.^27^ A previous study showed that DFCs can rescue the degeneration of inflamed periodontal ligament stem cells, suggesting a promising future in periodontal regeneration.^7^ In addition, DFCs can form root-like tissues upon being transferred into a treated dentin matrix scaffold in the alveolar fossa.^5^ DFCs have multiple differentiation competencies, they have low immunogenicity and are easily accessed. Given their remarkable properties, DFCs have been considered pivotal seed cells in periodontal tissue engineering and hopefully provide a novel approach to regeneration therapy.^7,28^

Previous studies have emphasized the accurate transfer of genetic information, which is essential for life. RNA Pol II can accelerate translation by the enzymatic machinery to transcribe protein-coding genes into mRNA.^29^ Transcriptional elongation is a vital step during gene transcription, and the SEC has a positive regulatory effect on this process.^22^

For the initial induction stage of transcription, an external stimulus is required. In mammals, the different SECs form in different combinations with common activators to regulate different genomes. SECs perform initiation and extension functions in gene transcription and can also modify the transcriptional region via histone methylation.^30^ The SEC is necessary in the stage of Pol II release from the proximal suspension of the gene promoter and transcription of HIV-1 gene activation.^22,31^ The recruitment of the SEC by genomes can occur specifically or non-specifically. Specific recruitment means that the SEC can be recruited to certain sites within the genome as directed by the DNA binding domain, and a particular recruitment factor can stabilize the structure of the SEC. Detection of non-specific recruitment at the genome-wide level shows that SEC binding is widespread in the genome, suggesting that the SEC also plays a corresponding role in the basic transcription process and that the mechanism might be related to transcriptional coactivators.^19,32^

The SEC is usually compromised of four subunits: a member from each of the AFF and ELL protein families, P-TEFb, and AF9 or ENL. The P-TEFb complex is a heterodimer that consists of CDK9 and CyclinT. It is essential for eukaryotic transcription and is necessary for most gene expression; its regulatory mechanism was first elucidated in HIV transcription. Moreover, Ser-2 and Thr-4 of the CTD, a heptapeptide structure, are phosphorylated by P-TEFb during transcription, providing the necessary conditions for transcriptional elongation.^22,33^ ELL2 is a functional transcription elongation factor that is able to accelerate the catalytic rate of transcription. RNA Pol II inhibition is prevented by the withdrawal of RNA Pol II from the 3’ end domain in the newly synthesized RNA on account of the activity of ELL2. At the same time, the half-life of ELL2 protein is remarkably improved after binding to AFF1 or AFF4, which can protect ELL2 from degradation.^34^ Moreover, demonstrating high homology, ENL and AF9 can help to achieve attachment between SEC and RNA Pol II by interacting with the RNA Pol II and becoming linked to the PAF1 complex (PAF1c). In addition, the YEATS region of ENL and AF9, a novel acetyl-binding module that is directly associated with histone acetylation and H3K79 methylation, is an indispensable structure involved in enhancing transcriptional stimulation.^35,36^

Formation of an SEC with AFF1 or AFF4 is a primary control factor in cancer pathogenesis and development. SEC-like 2 (SEC-L2) or SEC-like 3 (SEC-L3), containing factor AFF2 or AFF3, respectively, was also discovered through biochemical isolation. Among the SEC family members, AFF4-formed SECs play a primary role in cells after receiving rapid transcriptional induction and demonstrates high kinase activity of the polymerase II C-terminal domain in cells.^32,34,37^ Thus, SECs have complex combination modes with diverse internal variability. SECs, as a large family system, may have different mechanisms driving their regulation of gene transcription, which need further exploration.

By promoting the process of RNA elongation, the AFF family of proteins serve as transcriptional activators, and the family consists of four members.^20^ AFF1 and AFF4 originate from the same branch in the phylogenetic trees, while AFF2 and AFF3 belong to another branch. AFF1 and AFF4 are both known scaffold proteins in SECs, but they exist in different SECs, demonstrating primarily distinguishable gene target specificities. A previous study revealed that both AFF1 and AFF4 can efficiently activate HIV gene transcription, and the results of these functions were confirmed by analysis of the transcriptional synthesis efficiency of the SECs at the mRNA level. In detail, AFF1-formed SECs are more efficient when functioning the viral protein Tat, while AFF4-formed SECs are more potent upon heat shock under the induction of HSP70. The difference in their ability to activate early HIV transcription results from the different recruitment of viral promoters and their ability to interact with PAFc.^31^

AFF1 is a fusion partner that is strongly expressed in mixed-lineage leukaemia (MLL) and acute lymphoblastic leukaemia (ALL).^38^ AFF2, as an X-linked gene, and it has considerable expression in the placenta and brain. The repressed expression of AFF2 can result in intellectual disability and mental retardation, such as FRAXE.^39^ AFF3 is also involved in fusion activity with intellectual disability. The expression of AFF3 is high in lymphocytes, and it is upregulated in cortical neurons relative to other cell types.^40^

AFF4 contains a common conserved C-terminal homology domain that is also found in the rest of the AFF protein members, and it has an N-terminal region that interacts with other subunits of the SEC. Among its family members, AFF4 is the most widely distributed factor in human tissues, and it is associated with a variety of life activities.^32^ As the fused subunit of MLL chimaeras, AFF4 can bring transcription elongation factors to targeted leukaemic genes. In leukaemic cells, AFF4-depleted SEC can result in an obvious reduction in MLL chimaera target gene expression.^20^ AFF4 can activate HIV-1 proviral transcription and transcription elongation factors, which are dispersed along a flexible AFF4 scaffold and can be recruited by the HIV-1 protein Tat.^41^ In head and neck squamous cell carcinoma cells, AFF4 can improve tumour initiation and tumorigenesis capability though SOX2.^42^ Previous research found that Aff4/Af5q31-null mice exhibited azoospermia, suggesting a nonnegligible effect on Sertoli cells. Moreover, in regard to FRAXE, AFF4 remains functionally redundant during brain development.

To explore the effect of AFF4 on osteogenic differentiation of human DFCs, we first investigated its expression level and found that the expression of *AFF4* was the highest among the members of the *AFF* family. Then, the knockdown and overexpression of *AFF4* were achieved through siRNA and lentivirus treatment, respectively. *AFF4* depletion repressed the osteogenic differentiation ability of DFCs. Accordingly, the overexpression of *AFF4*, mediated by lentiviral treatment, promoted osteogenic differentiation.

For stem cells, differentiation into specialized cells requires specific cell signals and epigenetic regulation. Epigenetic changes, such as DNA methylation and histone modifications and changes, mainly occur in association with noncoding RNA expression and deregulation of epigenetic processes. They can adjust gene expression levels in the multistep process of carcinogenesis.^43^ During the differentiation course of stem cells, epigenetic regulation points to functionally correlative modifications within the genome, while the nucleotide sequence remains unchanged.^44^

As a 2-oxoglutarate and Fe^2+^-dependent hydroxylase,^45^ ALKBH1 functions as a demethylase for a new epigenetic modification of DNA N^6^-methyladenine (N^6^-mA), and provides dual effects regarding the central network of transcription factors regulating embryonic stem cells.^46,47^ ALKBH1 possesses high demethylation activity, indicating its effect on silencing epigenetic genes.^48^ It participates in damage reversal through oxidative demethylation of 1-methyladenine and 3-methylcytosine, and it is involved in the regulation of transcription mechanisms.^49^ ALKBH1 also has a prominent influence on epigenetic regulation by accommodating pluripotency markers and differentiation genes during embryogenesis. In embryonic stem cells, ALKBH1 is involved in the central network transcription factors regulating pluripotency. A deficiency in ALKBH1 delays the expression of pluripotency-related markers and postpones the induction of genes that participate in early differentiation.^45^ Our previous research also notes that ALKBH1 has a close relationship with the osteogenic differentiation of human MSCs and is well described as a factor in MSCs that promotes osteogenesis.^24^ Given these research facts, we hypothesized that ALKBH1 might be involved in the osteogenic differentiation mechanism of DFCs. Here, we found that ALKBH1 also functions as a stimulus for osteogenic differentiation of human DFCs under the regulation of *AFF4*.

In summary, our data indicate that AFF4 is highly expressed in DFCs. *AFF4* depletion impairs the osteogenic differentiation of DFCs. In contrast, the lentivirus-mediated overexpression of *AFF4* promotes osteogenic differentiation of DFCs. Mechanistically, AFF4 enhances *ALKBH1* transcription, producing a positive effect on the osteogenic differentiation of human DFCs. In this study, the results reveal that the scaffold protein AFF4, as a critical epigenetic regulator, plays an important role during the osteogenic differentiation of human DFCs. Our study on the molecular mechanism of AFF4 in regulating DFC osteogenic differentiation and its key role in determining the fate of stem cells can provide an important experimental basis for the clinical treatment of bone repair and reconstruction in periodontal diseases.

## Materials and methods

### Cell culture

Human DFCs were harvested from tooth buds of impacted third molars with undeveloped roots. Patients who underwent extractions for orthodontic reasons were 8–18 years old and had provided informed consent; there was no gender requirement. Procedures took place at the West China Hospital of Stomatology, Sichuan University. None of these selected patients had systemic disease, a history of radiotherapy, had undergone chemotherapy or had a history of smoking. All experimental procedures and protocols were approved by West China Hospital of Stomatology, Sichuan University.

Isolated follicle tissues were immediately transferred into phosphate-buffered saline (PBS) supplemented with 100 units mL^−1^ penicillin (Gibco) and 100 µg·mL^−1^ streptomycin (Gibco) once obtained. The human dental follicles were cut into approximately 1 mm^3^ tissue blocks and digested for 1 h at 37°C with agitation in PBS containing 3 mg·mL^−1^ type I collagenase (Gibco) and 3 mg·mL^−1^ dispase (Gibco).

The digested suspensions were transferred into 21 cm^2^ culture dishes and were cultured in alpha-minimum Eagle’s medium (α-MEM) (Gibco) supplemented with 20% foetal bovine serum (FBS) (Gibco), 100 units·mL^−1^ penicillin and 100 µg·mL^−1^ streptomycin. Culture dishes were incubated at 37 °C with 5% CO_2_, and the medium was changed every 2 days. The cells were passaged when the cell fusion reached 80%, as assessed under the microscope. The cells were then cultured with α-MEM supplemented with 10% FBS, 100 µg·mL^−1^ streptomycin and 100 units per ml penicillin, and they were passaged every 4–5 days. Passage 3 cells were chosen for subsequent experiments.

### Osteogenic induction

To induce osteogenic differentiation, the cells were grown in osteogenic medium containing 10 nmol·L^−1^ dexamethasone (Sigma), 50 μmol·L^−1^ ascorbic acid (Sigma) and 10 mmol·L^−1^ β-glycerophosphate (Sigma) when the cells reached 50%–60% confluence.

### Gene knockdown

For gene knockdown, we obtained a targeting control and an *AFF4* siRNA from Sangon Biotech (Shanghai, China). The experimental protocols were performed according to the manufacturer’s protocol.^50^ In brief, a transfection solution contained 50 nmol·L^−1^ Lipofectamine™ RNAiMAX in 200 µL of Opti-MEM® I Medium without serum was prepared and was incubated for 25 min at room temperature. When the cells reached 50%–70% confluence, they were diluted in complete growth medium without antibiotics, and the transfection solution with *AFF4*/control-siRNA was added and incubated with the cells for 10 h at 37 °C in a CO_2_ incubator. The efficiency of the gene knockdown was assessed. The sequence of the *AFF4*-siRNA is recorded in Table 1.

**Table 1:** The primer sequences for qRT-PCR and siRNA

Gene	Primers sequences
*AFF1*	F:AAAGGCCAGCATGGATCAGAA R:AAAGGCCAGCATGGATCAGAA
*AFF2*	F:CTGACAGCGAATCTAATGAGGC R:CATTGGTTGGATGATTGGAGGA
*AFF3*	F:GTCATCTCGTTGGAGTTCCCA R:AGTGCCTCTCTTACTCTGCTG
*AFF4*	F:AAAGGCCAGCATGGATCAGAA R:GTGATTTGGAGCGTTGATGTTC
*DLX5*	F:CTACAACCGCGTCCCAAG R:CTACAACCGCGTCCCAAG
*SP7*	F:CATCTGCCTGGCTCCTTG R:CAGGGGACTGGAGCCATA
*RUNX2*	F:GTGCCTAGGCGCATTTCA R:GCTCTTCTTACTGAGAGTGGAAGG
*BGLAP*	F:TGAGAGCCCTCACACTCCTC R:ACCTTTGCTGGACTCTGCAC
*GAPDH*	F:AGCCACATCGCTCAGACAC R:GCCCAATACGACCAAATCC
*ALKBH1*	F:CGCGAGATGGGGAAGATGGCA R:TCCAAGTCTCAGCTGTGAGG
For siRNA	
*AFF4*	F:GCAGCACTCAACTCAATCT R:AGAUUGAGUUGAGUGCUGCTT

*qRT-PCR* quantitative RT-PCR, *siRNA* small interfering RNA

### Gene overexpression

For overexpression experiments, lentiviruses expressing empty vectors GFP and *AFF4* were purchased from Genechem (Shanghai, China). The *AFF4*/control lentivirus infections were accomplished with Polybrene (Genechem). DFCs were cultured in 6-well culture plates to 30%–40% confluence, at which point they were infected with a lentivirus at a MOI = 10. After incubation for 72 h at 37 °C in a CO_2_ incubator, the DFCs were selected by treatment with puromycin (Sigma) at a concentration of 2.5 µg·mL^−1^ with continuous treatment until the cells were analysed for overexpression efficiency and then were used in subsequent experiments.

### RNA isolation and qRT-PCR

With reference to the product directions, all RNA in this experiment was extracted with TRIzol reagent (Invitrogen), and cDNA was generated from RNA via a PrimeScript RT regent kit (Takara, Dalian, China) and gDNA eraser (Takara, Dalian, China).

qRT-PCR was performed with SYBR Premix Ex Taq (Takara, Dalian, China) and an ABI real-time PCR system (Applied Biosystems, Foster City, CA). The housekeeping gene *GAPDH* was regarded as a standard for normalizing expression. Using the 2^−ΔΔCt^ method, the relative gene expression levels were demonstrated as the fold changes compared to the control. Thermocycling was performed as follows: step 1 (repeat 1) at 95 °C for 30 s, step 2 (repeat 40) at 95 °C for 5 s and 60 °C for 30–60 s, and step 3 dissociation. The primer sequences for qRT-PCR are listed in Table 1.

### Western blot

DFCs were lysed in 6-well plates with lysis buffer and protease inhibitor cocktail (Roche) on ice. To remove the cell debris, the cells were centrifuged for 15 min at 18 000 × *g* and 4 °C. The supernatants were obtained and heated for 5 min with sample buffer at 99 °C. The protein (15 µL per sample) was loaded for analysis.

Protein lysates were transferred onto PVDF membranes by a transfer apparatus (Bio-Rad),^51^ and α-tubulin was chosen as a control marker. After immersion in blocking buffer (5% skim milk) for 1.5 h, the membranes were incubated with rabbit anti-AFF4 (Abcam, 1:1 500), rabbit anti-ALKBH1 (Abcam, 1:1 000), rabbit anti-α-tubulin (Sigma, 1:2 000), or rabbit anti-Flag (Sigma, 1:1 000) overnight at 4 °C, which was followed by incubation with a secondary antibody for 1 h at room temperature. Then, the membranes were incubated with SuperSignal reagents (Pierce, Rockford, IL), and the antibody-antigen complexes were obtained. The semiquantification of protein levels was analysed by ImageJ software.

### ALP staining and activity

DFCs underwent osteogenic induction in 24-well culture plates. After culturing with osteogenic differentiation medium for 7 days, the cells were collected for ALP staining. They were washed with PBS 3 times and then were fixed with paraformaldehyde.^52^ Then, the cells were incubated with an ALP staining solution in the dark for 40 min; the solution was prepared from a BCIP/NBT Alkaline Phosphatase Color Development kit (Beyotime Biotechnology, Shanghai, China).

ALP activity was calculated with a Alkaline Phosphatase Assay kit (Beyotime Biotechnology, Shanghai, China) and BCA Protein Assay kit (Beyotime Biotechnology, Shanghai, China) in accordance with the manufacturer’s protocols. Protein samples were diluted with 0.5 mmol·L^−1^*p*-nitrophenyl phosphate from the Alkaline Phosphatase Assay kit. The BCA protein concentration was evaluated with absorbance measurements at 562 nm with a standard calcium curve.

### ARS staining

After induction with osteogenic medium in 24-well culture plates for 3 weeks, DFCs were prepared for evaluation of mineralization ability. After washing with PBS 3 times, the DFCs were fixed with 4% paraformaldehyde for 25 min and then stained with alizarin red S solution (Solarbio Science & Technology, Beijing, China) in the dark for 30 min.

### Quantitative analysis of calcium concentration

When alizarin red S staining was finished, mineralized bone nodules were treated with 10% cetylpyridinium chloride for 15 min, which was dissolved in PBS. The quantitative analysis of calcium concentration was evaluated by collecting measurements at 562 nm with a standard calcium curve.

### Chromatin immunoprecipitation (ChIP) assay

In accordance with the manufacturer’s instructions, a ChIP assay was conducted via an EZ-Zyme Chromatin Prep kit (Millipore) and Magna Chip HiSens (Millipore) using either a normal rabbit IgG (Abcam) as a control or an antibody against AFF4 (Abcam) for the experimental group. First, the DNA-protein complexes were dissociated. Then, the pulled-down DNA as well as the input DNA were tested by qRT-PCR analysis, and the qRT-PCR primers were designed to identify the target promoter regions. The ChIP assay results are demonstrated in relation to the input DNA.

### Statistical analysis

All data are presented as the mean ± SE. Statistical differences were performed via unpaired two-tailed Student’s *t* test for comparisons between two groups or by one-way ANOVA followed by Tukey’s post hoc test for multiple comparisons. A *P* value of < 0.05 was regarded as statistically significant.
